# Single-step approach to sensitized luminescence through bulk-embedded organics in crystalline fluorides

**DOI:** 10.1038/s42004-020-00410-0

**Published:** 2020-11-10

**Authors:** Per-Anders Hansen, Tomas Zikmund, Ting Yu, Julie Nitsche Kvalvik, Thomas Aarholt, Øystein Prytz, Andries Meijerink, Ola Nilsen

**Affiliations:** 1 Department of Chemistry, University of Oslo, Oslo, Norway; 2 Institute of Physics, Academy of Sciences of the Czech Republic, Prague, Czech Republic; 3 Debye Institute for NanoMaterials Science, Utrecht University, Utrecht, The Netherlands; 4 Department of Physics, University of Oslo, Oslo, Norway

**Keywords:** Organic-inorganic nanostructures, Inorganic LEDs, Optical materials

## Abstract

Luminescent materials enable warm white LEDs, molecular tagging, enhanced optoelectronics and can improve energy harvesting. With the recent development of multi-step processes like down- and upconversion and the difficulty in sensitizing these, it is clear that optimizing all properties simultaneously is not possible within a single material class. In this work, we have utilized the layer-by-layer approach of atomic layer deposition to combine broad absorption from an aromatic molecule with the high emission yields of crystalline multi-layer lanthanide fluorides in a single-step nanocomposite process. This approach results in complete energy transfer from the organic molecule while providing inorganic fluoride-like lanthanide luminescence. Sm^3+^ is easily quenched by organic sensitizers, but in our case we obtain strong fluoride-like Sm^3+^ emission sensitized by strong UV absorption of terephthalic acid. This design allows combinations of otherwise incompatible species, both with respect to normally incompatible synthesis requirements and in controlling energy transfer and quenching routes.

## Abstract (web summary)

Aromatic molecules often afford strong and tunable absorption, desirable for lighting applications, but incorporating them in inorganic structures without luminescence quenching is challenging. Here the broad absorption from terephthalic acid is combined with inorganic fluoride-like lanthanide luminescence in a composite material.

## Introduction

Luminescent materials based on lanthanides are a well-developed field with respect to materials^1^, physical mechanisms^2^ and use in applications like lighting^3^, laser^4^, optoelectronics^5^ and solar cells^6–8^. An increasing amount of research is focused on advanced optical systems, requiring interactions between two or more different types of ions. Examples are downconversion^9^ and upconversion^10,11^. There are two major bottlenecks slowing down this development. (1) The dopants must be controlled on a (sub-)nanometre scale to prevent unwanted cross-relaxations and other quenching processes while at the same time optimizing the desired energy transfers, and (2) combining the weak narrow line-absorbing lanthanides with a strong broad band-absorbing and tunable sensitizer.

Twenty years ago, the term downconversion was coined^12^ and an efficiency of 190% was demonstrated to be possible. Four years later, an efficiency of 194% was shown in BaF_2_:Gd^3+^,Eu^3+ ^^13^. Recently, record high upconversion efficiencies were realized in multi-shell nanoparticles of NaGdF_4_:Nd^3+^,Yb^3+^,Tm^3+ ^^14^. Common for both cases is the use of crystalline fluoride host matrices and multiple lanthanides. For materials using more than two different optically active ions, it is beneficial to separate these in a multi-shell/layered structure so that only two ions can interact at a time, preventing cross-relaxation and quenching. The material systems demonstrated so far that both allow multi-shell designs and can incorporate trivalent lanthanides is limited, and the majority of literature revolve around NaYF_4_-type fluorides. Another common factor for downconversion and upconversion is that these processes are difficult to combine with a strongly absorbing sensitizer. One reason is that there are limited options of possible ions that easily fit into the cation sites in these crystalline fluorides. Recently, Fischer et al. showed that, even though Ce^3+^ cannot normally be used as a ultraviolet (UV) sensitizer for Eu^3+^ due to a quenching Ce^3+^–Eu^3+^ charge transfer state, they were successful in avoiding this quenching mechanism by separating Ce^3+^ and Eu^3+^ in separate layers and using Tb^3+^ ions to allow energy migration from Ce^3+^ to Tb^3+^ to Eu^3+^ while spatially separating Ce^3+^ and Eu^3+^ in core–shell TbF_3_ nanoparticles^15^. It is still a challenge to find suitable sensitizers as the number of possible sensitizer ions that easily fit into TbF_3_ and similar matrices is limited. Aromatic molecules belong to a material class that show excellent sensitizing abilities, very strong absorption and full tunability of the absorption range^16^. One solution is to anchor such organic sensitizer molecules on fluoride nanoparticles, but also this is challenging without causing concentration quenching in the organics. An easy, controllable way of combining strongly absorbing species with multilayered crystalline fluorides is still lacking.

Using atomic layer deposition (ALD), we have previously shown that multilayered structures of luminescent oxides with layer thicknesses <0.4 nm can be synthesized^17^, in addition to multilayer structures with multi-lanthanide incorporation^18,19^. Deposition of nearly all lanthanide oxides can similarly be realized by ALD^20^, and organic molecules can relatively easily be incorporated into solid films^21^. ALD is also called molecular layer deposition when including organic molecules. Lanthanide fluorides have been successfully deposited by ALD at low (175 °C) temperatures^22^, minimizing the risk of thermal decomposition when incorporating organic components. ALD is in fact well suited for all the different components in organic sensitized multi-component luminescent systems.

**Fig. 1:**
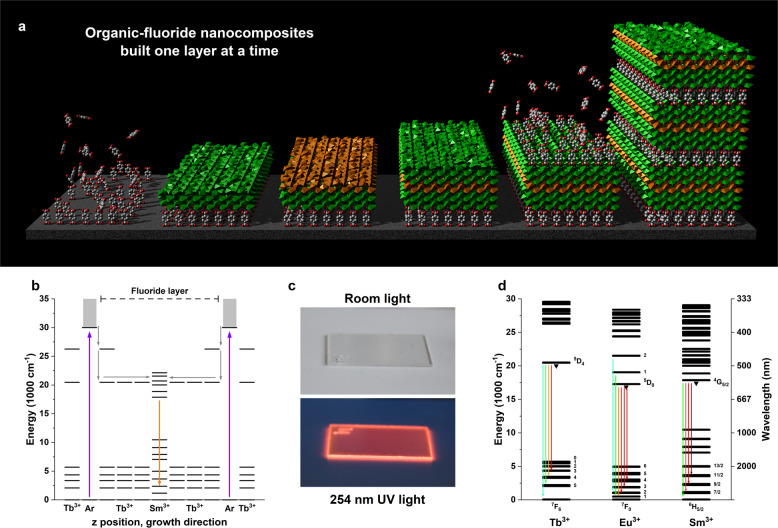
Overview of the sample design and energy levels. **a** Illustration of how the nanocomposite film is built up layer by layer. Terephthalic acid molecules are shown attaching to the surface, while TbF_3_ and SmF_3_ layers are shown in green and orange, respectively. **b** Energy-level diagram of the sample design, showing UV absorption in the aromatic acid (Ar), energy transfer to Tb^3+^, migration towards Sm^3+^ and finally Sm^3+^ emission. Note that some energy levels have been omitted for clarity. **c** Photograph of the 100Sm sample on a silica substrate in room light (top) and 254 nm UV light (bottom). **d** Energy-level diagram of Tb^3+^, Eu^3+^ and Sm^3+^, including names of the energy levels involved in radiative transitions. These transitions are shown with arrows with colours corresponding to the emitted photon.

Here we combine the known ALD chemistries of metal fluorides from metal-thds (thd = 2,2,6,6-tetramethyl heptanedione) and NH_4_F^23^ with strongly UV-absorbing aromatic acid-based hybrid materials^24^ to combine the conversion and luminescence properties of multilayer crystalline lanthanide fluorides with the strong absorption strength and sensitization of organics. We successfully combine two different material classes and obtain a molecule-doped crystalline multilayer film structure while also preventing quenching of the phonon-sensitive Sm^3+^ by the high-energy phonons on the acid. The nanocomposite structure is illustrated in Fig. 1, along with an energy-level diagram to show the energy flow, a photo of a sample and an energy-level diagram showing the emission lines from the lanthanides included in this work. The essence is that by combining organics and inorganic fluorides in a nanocomposite, we have achieved a combination of very strong absorption and highly efficient luminescence that would not be possible in either the organic or fluoride alone. In addition, embedding organic sensitizers into the bulk of inorganic materials also overcomes the often encountered photobleaching, as the molecules are now protected from (photo)chemical degradation by air or moisture.

This layer-by-layer nanocomposite design is generalizable for combinations of other optical material classes as well. ALD is capable of depositing nearly the entire periodic table including most inorganic material classes like oxides, sulfides, nitrides, fluorides and phosphates^25^ in addition to organic–inorganic hybrid and purely organic materials^26^. In the recent years, several different combinations of material classes have been synthesized as nanocomposites by ALD. This includes multiple different oxides^27,28^, oxide–nitride^29^, oxide–sulfide^30^, oxide–metal^31,32^, oxide–hybrid^33^, and even controlled crystalline oxide–amorphous oxide^34^ nanocomposites. Several of these are used to control optical properties^30,34^ or in optics^31^. ALD is a powerful synthesis tool allowing very complex designs that also includes two or more material classes. With this in mind, it is our opinion that there is large unexplored terrain within advanced optical materials that can and should be investigated with ALD.

## Results

### ALD growth

**Fig. 2:**
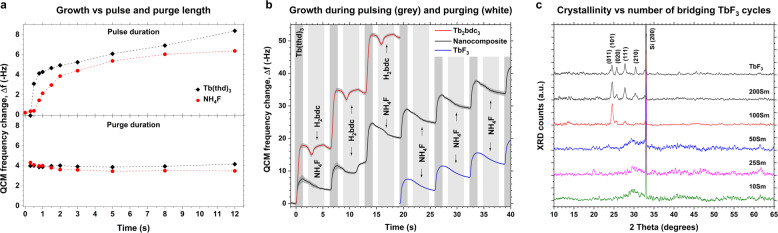
ALD growth and crystallinity. **a** QCM frequency change per ALD cycle as a function of pulse and purge parameters. **b** QCM frequency change during ALD cycle sequences of Tb_2_bdc_3_, TbF_3_ and a 4Sm nanocomposite. Tb(thd)_3_ pulses are marked in dark grey, H_2_bdc and NH_4_F anion pulses are marked in light grey and nitrogen purges are marked in white. An increase and decrease in the graph indicates mass gain and reduction, respectively. **c** XRD data of *x*Sm films in addition to a deposition of 2000 cycles TbF_3_.

Fluoride depositions with ALD is by far not as well explored as oxide depositions, and indeed lanthanides fluorides have not been deposited before using the Ln(thd)_3_ and NH_4_F precursor pairs. Exploring this ALD chemistry in detail is outside the purpose of this work. Nonetheless, for controlled growth of our multilayer design, we explored the saturation conditions for LnF_3_ cycles, Fig. 2a. Despite clear indications of ALD growth in the initial 1 and 2 s for the cation and anion, respectively, the growth did not saturate completely for longer pulse times up to 12 s. We also found that the growth rate had a large dependence on how much NH_4_F was loaded in the precursor boat, with growth rate per cycle (GPC) varying between 11 and 21 pm/cycle. In addition, there was always a gradient of 5–20% between samples spaced 8 cm apart along the gas flow direction. Nevertheless, increasing any pulsing or purging parameters longer than 1.5/1/3/1 s had negligible effect on both growth rate and gradients. Based on this, we decided to use 1.5/1/5/1 as pulsing and purging parameters for all cycles. Note that all quartz crystal microbalance (QCM) results use 1.5/1/3/1 as standard parameters.

For the aromatic–lanthanide hybrid Ln_2_bdc_3_ cycles (bdc = benzenedicarboxylate), it was found that the parameter 1.5/1/2/1 resulted in very even films throughout the reaction chamber and was found to be sufficient for proper surface saturation with a GPC of 175.7 pm/cycle with a thickness difference of 0.6% between samples 8 cm apart. We also experience that these hybrid depositions give identical results between different ALD reactors of the same type. Thus we did not investigate the growth chemistry of the hybrid cycles further. Nevertheless, it was decided to use longer H_2_bdc pulses (5 s, as for NH_4_F) to ensure saturation also when combining Ln_2_bdc_3_ cycles with LnF_3_ cycles.

To investigate whether and how combining hybrid Ln_2_bdc_3_ and fluoride LnF_3_ cycles affect each other’s growth, a multilayer deposition consisting of one Ln_2_bdc_3_ and nine LnF_3_ cycles was investigated with QCM, Fig. 2b. This represents a 4Sm sample according to the naming convention used here. In addition, pure TbF_3_ and pure Tb_2_bdc_3_ cycles are shown for comparison. Each of the three data sets is averaged over 16 cycles for 4Sm (named Nanocomposite in Fig. 2b) and Tb_2_bdc_3_, and 80 cycles for TbF_3_. For the pure Tb_2_bdc_3_ and TbF_3_ cycles, three identical cycles are plotted after each other to illustrate the continuous growth. For the nanocomposite, only five of the nine TbF_3_ cycles are shown in addition to the Tb_2_bdc_3_ cycle.

The LnF_3_ cycles do not show any significant changes after the Ln_2_bdc_3_ cycle, indicating that the hybrid cycle does not change the total mass deposited during each LnF_3_ cycle. On the other hand, it can be seen that the H_2_bdc pulse causes a mass gain for the following Tb(thd)_3_ pulse. During the H_2_bdc pulse, there is an initial mass decrease. This is rapidly followed by a similar sized mass increase for pure Tb_2_bdc_3_ and a delayed mass increase for the nanocomposite.

There are several possible surface reactions during these nanocomposite depositions. The following reactions show the possible reactions between |-Tb(thd)_*x*_ (*x* = 1 or 2) species on the surface and H_2_bdc and HF (from NH_4_F) anion pulses. Ideal reactions would be a simple ligand exchange mechanism, releasing Hthd as a leaving molecule. It is also possible that H_2_bdc can etch TbF_3_ surfaces as H_2_bdc is a stronger acid than HF. The opposite etching of Tb_2_bdc_3_ by HF is less likely. Lastly, it is possible for the bdc^2−^ anion to bond to two different Tb^3+^ ions, creating a “bridge” between them. All of these reactions during the anion pulse will cause a mass decrease, apart from the etching reaction. The mass difference Δ*m* is calculated with respect to one mole Tb^3+^ surface species. For comparison, the masses of Tb^3+^, thd^−^, F^−^ and bdc^2−^ are 159, 183, 19 and 164 g/mol, respectively.

Ideal reactions

1 $$\begin{array}{l}| \mbox{-} {\mathrm{Tb}}\left( {{\mathrm{thd}}} \right)_{\it{x}} + {\mathrm{HF}}\left( {\mathrm{g}} \right) \to | \mbox{-} {\mathrm{Tb}}\left( {{\mathrm{thd}}} \right)_{{\it{x}}{\mathrm{ - 1}}}{\mathrm{F}} + {\mathrm{Hthd}}\left( {\mathrm{g}} \right)\\ \Delta m_{\mathrm{1}} = - {\mathrm{164}}\,{\mathrm{g/mol}}\end{array}$$

2 $$\begin{array}{l}| \mbox{-} {\mathrm{Tb}}\left( {{\mathrm{thd}}} \right)_{\it{x}} + {\mathrm{H}}_{\mathrm{2}}{\mathrm{bdc}}\left( {\mathrm{g}} \right) \to | \mbox{-} {\mathrm{Tb}}\left( {{\mathrm{thd}}} \right)_{{\it{x}}{\mathrm{ - 1}}}\left( {{\mathrm{Hbdc}}} \right) + {\mathrm{Hthd}}\left( {\mathrm{g}} \right)\\ \Delta m_{\mathrm{2}} = - {\mathrm{18}}\,{\mathrm{g/mol}}\end{array}$$

Etching reactions

3 $$\begin{array}{l}| {\mbox{-}} {\mathrm{TbF}}_{\it{x}} + {\mathrm{H}}_{\mathrm{2}}{\mathrm{bdc}}\left( {\mathrm{g}} \right) \to | {\mbox{-}} {\mathrm{TbF}}_{{\it{x}} - {\mathrm{1}}}\left( {{\mathrm{Hbdc}}} \right) + {\mathrm{HF}}\left( {\mathrm{g}} \right)\\ \Delta m_{\mathrm{3}} = + {\mathrm{146}}\,{\mathrm{g/mol}}\end{array}$$

Bridging reactions

4 $$\begin{array}{l}{\mathrm{2}}| {\mbox{-}} {\mathrm{Tb}}\left( {{\mathrm{thd}}} \right)_{\mathrm{2}} + {\mathrm{H}}_{\mathrm{2}}{\mathrm{bdc}}\left( {\mathrm{g}} \right) \to \\ {\mathrm{2}}| {\mbox{-}} {\mathrm{Tb}}\left( {{\mathrm{thd}}} \right)\left( {{\mathrm{bdc}}} \right)_{{\mathrm{0}}{\mathrm{.5}}} + {\mathrm{2Hthd}}\left( {\mathrm{g}} \right)\\ \Delta m_{\mathrm{4}} = - {\mathrm{101}}\,{\mathrm{g/mol}}\end{array}$$

5 $$\begin{array}{l}{\mathrm{2}}| {\mbox{-}} {\mathrm{Tb}}\left( {{\mathrm{thd}}} \right)\left( {{\mathrm{bdc}}} \right)_{{\mathrm{0}}{\mathrm{.5}}} + {\mathrm{H}}_{\mathrm{2}}{\mathrm{bdc}}\left( {\mathrm{g}} \right) \to {\mathrm{2}}| {\mbox{-}} {\mathrm{Tb}}\left( {{\mathrm{thd}}} \right)\left( {{\mathrm{Hbdc}}} \right)\\ \Delta m_{\mathrm{5}} = + {\mathrm{83}}\,{\mathrm{g/mol}}\end{array}$$

6 $$\begin{array}{l}{\mathrm{2}}| {\mbox{-}} {\mathrm{Tb}}\left( {{\mathrm{thd}}} \right)\left( {{\mathrm{bdc}}} \right)_{{\mathrm{0}}{\mathrm{.5}}} + {\mathrm{3H}}_{\mathrm{2}}{\mathrm{bdc}}\left( {\mathrm{g}} \right) \to \\ {\mathrm{2}}| {\mbox{-}} {\mathrm{Tb}}\left( {{\mathrm{Hbdc}}} \right)_{\mathrm{2}} + {\mathrm{2Hthd}}\left( {\mathrm{g}} \right)\\ \Delta m_{\mathrm{6}} = + {\mathrm{65}}\,{\mathrm{g/mol}}\end{array}$$

Among these reactions, only Eq. 4 can provide an overall mass gain during the anion pulse. In the case of a bridging bdc^2^^−^ molecule, the overall mass gain is negative even though Eqs. 5 and 6 show positive mass gains. This is because the bridging reactions (Eq. 4) are followed by the bridge-opening reaction (Eq. 5) and ideal reactions (Eq. 2) removing the last thd^−^ groups. Bridge-opening and ideal thd reactions are shown in Eq. 6, showing the mass gain in this step is smaller than the mass reduction during the initial bridging reaction (Eq. 4). This bridging mechanism will then cause a temporary mass decrease followed by a mass increase while still maintaining a small overall mass decrease from start to finish. Only the etching reaction (Eq. 3) will cause a net mass gain.

Figure 2c shows the X-ray diffraction (XRD) patterns of the deposited *x*Sm films in addition to a deposition of 2000 cycles TbF_3_. It is seen that the films are amorphous for *x* ≤ 50 and crystallize as orthorhombic TbF_3_ (PDF# 32-1290) for larger *x* values.

### Optical properties

**Fig. 3:**
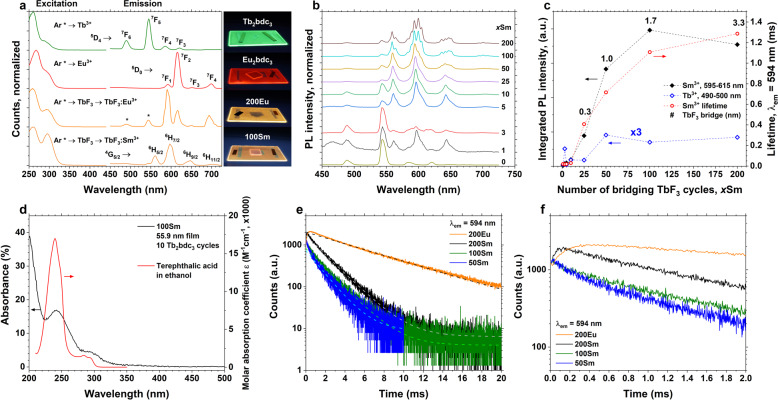
Photoluminescence characterization. **a** PL and PLE spectra of the four selected samples: Tb_2_bdc_3_, Eu_2_bdc_3_, 200Eu, and 100Sm. PL was recorded using a 280-nm diode while PLE was recording while monitoring the highest peak in each spectrum. Photographs show these samples under 254 nm UV light. The two 200Eu peaks marked asterisk (*) is likely Tb^3+^ emission. **b** Luminescence spectra of *x*Sm samples and Tb_2_bdc3, normalized to the most intense peak. Note the difference in 560 and 600 nm peaks between 25Sm and 100Sm. **c** PL intensity integrated over the 595–615 nm range (Sm^3+^, black, left axis), 540–550 nm (Tb^3+^, green, left axis) and lifetime of the 594 nm Sm^3+^ emission (red, right axis). Noted that the Tb^3+^ integrated intensity has been multiplied by 3 for clarity. The estimated thickness (in nm) of TbF_3_ separating Sm^3+^ and the organic molecules is given in numbers over the data points. **d** UV-Vis absorbance data from 100Sm on silica substrate; literature data of the molar absorption coefficient of terephthalic acid for comparison. **e** Decay of the ^4^G_5/2_ → ^6^H_7/2_ and ^5^D_0_ → ^7^F_1_ transitions of *x*Sm and 200Eu. An initial rise in the emission is seen for 200Sm and 200Eu only. **f** Decay lifetime modelling of these transitions using a single exponential function.

Figure 3a shows the photoluminescence (PL) and photoluminescence excitation (PLE) spectra of four selected samples. These are Tb_2_bdc_3_ showing Tb^3+^ emission, Eu_2_bdc_3_ showing red ^5^D_0_ → ^7^F_2_-dominated emission, 200Eu showing orange ^5^D_0_ → ^7^F_1_-dominated emission and 100Sm showing Sm^3+^ emission. Sm_2_bdc_3_ gives no detectable emission in the 200–1100 nm range upon 280 nm excitation. The 200Eu sample show two additional peaks (marked *) that are at too high energies to originate from ^5^D_0_. Based on their peak positions matching those of Tb^3+^, these are likely Tb^3+^ emissions. The PL is obtained using a 280-nm diode while PLE spectra are recorded monitoring the highest emission peak for all samples. All the samples clearly show the two excitation bands of the aromatic molecule.

Figure 3b shows normalized PL spectra of the *x*Sm samples. In addition, Tb_2_bdc_3_ is included to show the Tb^3+^ emission peaks as some Tb^3+^ emission is seen in all *x*Sm samples. Note that the total emission intensity of samples with *x* ≤ 10 is very low (see Fig. 3c). The 100Sm and 25Sm spectra show clear differences in the number and sharpness of the peaks, while 50Sm shows a superposition of these two spectra (most clearly seen by the additional hump around 600 nm).

**Fig. 4:**
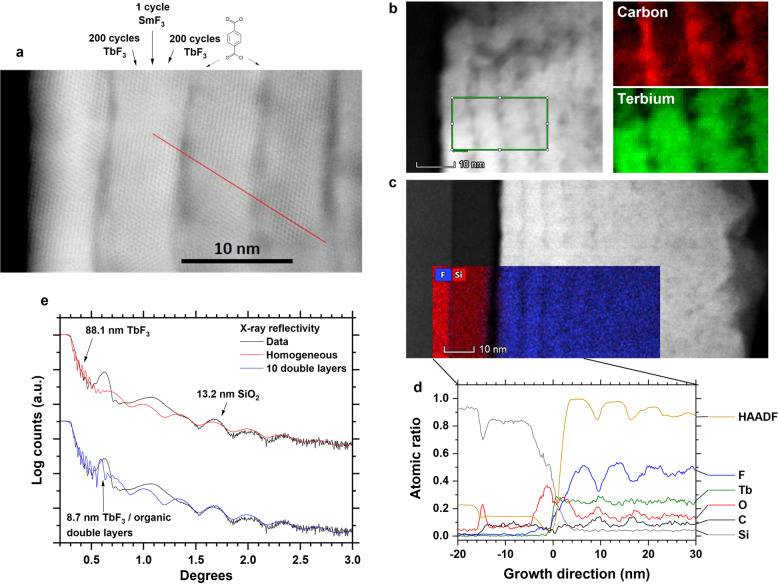
STEM and XRR characterization of the multilayer structure and composition. **a** STEM image showing the multilayer structure of 200Sm. The thin, darker lines are aromatic layers. The red line is an eye guide, showing a continuous crystal structure through two aromatic layers. Note that 401 LnF_3_ cycles results in about 18 cation layers between each organic layer. **b** STEM core-loss map showing thin layers that are carbon rich (red) and terbium deficient (green). **c** STEM EDS map showing thin fluorine-deficient layers. **d** Line scan showing the compositional changes along the growth direction. **e** XRR data of 200Sm, showing 3 distinct features; rapid oscillations from the thick total film thickness, long wave oscillations from the SiO_2_ layer and two non-sinusoidal peaks stemming from the multilayer structure.

Figure 3c shows the PL intensity of one Sm^3+^ and one Tb^3+^ peak integrated over the 595–615 and 540–550 nm range, respectively, and the PL lifetime of Sm^3+^ emission at 594 nm, as a function of TbF_3_ cycles separating the Tb_2_bdc_3_ and SmF_3_ cycles. As there is quite a bit of overlap between the Sm^3+^ and the weaker Tb^3+^ emission peaks, these ranges were chosen. An estimate of the TbF_3_ layer thickness (in nm) separating Sm^3+^ and the organic molecules is given in numbers over the data points. This estimate is calculated by taking the total film thickness (of the stack of 10 supercycles) from ellipsometry, substracting 10 nm for the 10 Tb_2_bdc_3_ layers, which are about 1-nm thick according to Fig. 4a, and dividing by 2 as there are 20 bridging layers (one on each side of Sm^3+^, Fig. 1b). Both the PL intensity and the PL lifetime of Sm^3+^ increase as the number of TbF_3_ cycles increases, showing that the emission intensity is dominated by the quenching rate of Sm^3+^. At a TbF_3_ layer thickness of 100 cycles, the intensity reaches a maximum while the lifetime still increases for 200Sm. This could indicate that not all excited states manage to migrate all the way to Sm^3+^ at 200 TbF_3_ cycle layer separating the aromatic molecule and Sm^3+^ ion. The integrated Tb^3+^ peak intensity is small until 50Sm, indicating that at this thickness the migration starts to become incomplete and some of excited Tb^3+^ ion will start to emit light. The apparent plateau for 50Sm, 100Sm and 200Sm is surprising. One possible explanation is that the Tb^3+^ emission, which is a balance between the rates for radiative emission on one side and migration and transfer to Sm^3+^ on the other side, is more affected by the local symmetry than the Sm^3+^ emission, which is not depopulated by migration or transfer to another ion. The 50Sm and 100Sm samples marks the difference between amorphous and (partly) crystalline TbF_3_, meaning that the ratio between high emission and transfer rates of 50Sm could be similar to the ration between lower emission and transfer rates for 100Sm. 200Sm has a higher Tb^3+^ emission than 100Sm, also supporting that increasingly incomplete transfer to Sm^3+^ is the reason for decreasing intensity and increasing lifetime of the Sm^3+^ emission between 100Sm and 200Sm. However, the Tb^3+^ emission of 3Sm is substantially higher than what should be expected from this explanation. Thus the apparent plateau might be caused by random variations of the samples.

Figure 3d shows the UV–visible absorbance of 100Sm sample deposited on silica substrate. The molar absorption coefficient, or epsilon value, of terephthalic acid dissolved in ethanol is shown for comparison^35,36^. The 100Sm film show two absorption peaks at approximately 250 and 300 nm that correspond well with the absorption spectrum of terephthalic acid. The absorption peaks also correspond well with the excitation profiles in Fig. 3a. The nanocomposite samples all contain exactly 10 Ln_2_bdc_3_ cycles. It is remarkable to obtain an emission spectrum from a fluoride lattice with over 15% absorbance in a thin film only 55.9 nm thick.

Figure 3e, f shows the PL decay of the most intense emission of 50Sm, 100Sm, 200Sm (^4^G_5/2_ → ^6^H_7/2_) and 200Eu (^5^D_0_ → ^7^F_1_). All decay curves can be fitted with a single exponential decay function. For both 200Sm and 200Eu, a small initial rise can be seen, indicating that there is some delay before the excited state energy generated in the aromatic acids reaches the Sm^3+^ and Eu^3+^ ions. For 200Eu, the rise is slower than for 200Sm. The slower rise in 200Eu indicates that the transfer from Tb^3+^ to Eu^3+^ is slower than Tb^3+^ to Sm^3+^, which could lead to a higher degree of incomplete transfer in the 200Eu case and more Tb^3+^ emission is expected. This combined with the matching peak positions makes it plausible that the two higher energy emission peaks for 200Eu in Fig. 3a are Tb^3+^ emissions.

### Multilayer and nanocomposite structure

Figure 4a show a scanning transmission electron microscopy–high-angle annular dark field (STEM-HAADF) image of the 200Sm sample. The sample structure is also illustrated above. The bright fluoride layers are about 7 nm thick, separated by darker organic layers. The red line highlights crystalline planes that pass through several organic layers. This could indicate that the organic molecules do not form a dense layer, as in the illustration in Fig. 1a, but rather are separated molecules as inclusions between the fluoride layers that have the possibility to continue the growth around them.

To investigate the chemical composition of these layers, core-loss and energy-dispersive X-ray spectroscopy (EDS) was used to map the carbon, terbium and fluorine contents, Fig. 4b, c. It is clear that the dark thin layers are rich in carbon and deficient in terbium and fluorine. In Fig. 4d, it is also seen that the oxygen level follows the same trend as carbon, showing peaks at the thin, dark layers. Although deficient in fluorine, it is also evident that the fluorine content only decrease by about 50% in these layers, further supporting that these dark layers contain both the aromatic acid and TbF_3_, which allows crystalline TbF_3_ to grow uninterrupted alongside the deposited bdc molecules.

To investigate the nanocomposite structure further, X-ray reflectivity (XRR) was performed on all samples. XRR data for 200Sm is shown in Fig. 4e, where three features can be seen. The top model (red) show a simple sample model consisting of one uniform 88.1 nm film on top of a 13.2 nm SiO_2_ layer. This model reproduces the rapid oscillations at small angles and long oscillations at large angles but not the two non-sinusoidal peaks in the middle. The second model is similar to the first, but the uniform film is replaced by 10 supercycles of 7.7 and 1.0 nm of high and low electron density materials. This model also reproduces the first of the two middle peaks to some extent, indicating that these two peaks stems from the multilayer structure. We were not able to produce a model that properly reproduced all the features at the same time. One explanation of this is that the sample is likely somewhat inhomogeneous with respect to crystallinity, which can be clearly seen in Fig. 4a. This will also affect the film thicknesses in each superlayer and in particular the crystallinity will affect the roughness between each layer which XRR is very sensitive towards.

## Discussion

### Film growth

Combining LnF_3_ and Ln_2_bdc_3_ cycles on the (sub)nanometre scale was successful for all LnF_3_/Ln_2_bdc_3_ cycle ratios. The crystallinity changes around a ratio of 201, i.e. the 100Sm sample where each Ln_2_bdc_3_ cycles is separated by 100 TbF_3_ + 1 SmF_3_ + 100 TbF_3_ cycles. The difference between 50Sm and 100Sm is clear both from XRD in Fig. 2c and the PL spectra in Fig. 3b. XRD shows that all samples with *x* < 100 is amorphous while *x* ≥ 100 shows reflections from orthorhombic TbF_3_ similar to the reflections seen for deposition of pure TbF_3_. The PL emission spectra show a clear difference in peak shapes, indicating a symmetry change around the Sm^3+^ ion for compositions between 50Sm and 100Sm. This agrees well with the onset of crystallinity. Deposition of pure TbF_3_ at 250 °C results in polycrystalline films, and, not surprisingly, adding the very different Ln_2_bdc_3_ cycle in between LnF_3_ cycles interrupts such crystal formation. A sufficiently thick layer is constructed for *x* > 100 to regain crystal growth, while also maintaining this across the bdc^2−^ dopant layer.

The STEM image of 200Sm in Fig. 4a also shows clear crystallinity and the LnF_3_ crystallites are larger than the LnF_3_ layer thickness and spans several organic layers. The only way such large crystallites can form is if the aromatic acid molecules only occupies a fraction of the possible surface sites. In this case, following LnF_3_ cycles can continue the crystalline growth uninterrupted between the organic molecules. This is partly shown in Fig. 4b where the level of carbon is out of phase with the level of fluorine. However, the fluorine level only decreases to about 50%. As each bdc^2−^ contains 8 carbon atoms and the level of carbon is only about 20%, there should be space between each molecule. Similar results with crystallites growing past organic layers have been obtained in ZnO-zincone multilayers deposited by ALD, using 1,4-dihydroxybenzene as the organic molecule^37^. Also in this case it did seem like there was sufficient room between the organic molecules to continue the growth of inorganic crystals.

The QCM in Fig. 2b show an unusual response with an initial mass decrease before an overall increase during the H_2_bdc pulses for both pure Tb_2_bdc_3_ and nanocomposite depositions. A detailed analysis of the growth mechanics is outside this work, but we can offer a possible explanation. When H_2_bdc is pulsed it reacts with |-Tb-thd_*x*_ surface species, producing Hthd as a leaving molecule and at least one Tb-bdc bond. This is a net mass decrease of only 10%. However, each H_2_bdc molecule can react with two |-Tb-thd_*x*_ sites, producing 2 Hthd molecules and a net mass decrease of 55%. As the H_2_bdc is about 7 Å long, this probably involves two different |-Tb-thd_*x*_ sites. Such bridging |-Tb-bdc-Tb-| species can further react with an additional H_2_bdc and produce two |-Tb-bdc-H species. This will in turn restore the net mass decrease to 10% as compared to the initial thd terminated surface. Such an approach can explain the initial mass decrease followed by a delayed mass increase back to about the same initial level but also implies that the kinetics of these reactions are significantly different. Our group have previously shown that the growth of metal–bdc hybrids can be affected by co-pulsing with other carboxylic acids, meaning that one metal–acid bond can be replaced with another similar acid^38^.

For the nanocomposite case on the other hand, the surface |-Tb-thd_*x*_ species are likely too far apart. Using the growth rate of 21 pm/cycle for TbF_3_ cycles and the unit cell dimensions of orthorhombic TbF_3_, each cycle adds about 0.42 Tb atoms per nm^2^ per cycle. On average, this is too far apart to bridge with a single H_2_bdc molecule. The mass increase during Tb(thd)_3_ pulses is larger for Tb_2_bdc_3_ than TbF_3_ depositions, also supporting that there are fewer reactive |-Tb-thd_*x*_ sites after the Tb(thd)_3_ pulse in TbF_3_ cycles. The mass increase during the first Tb(thd)_3_ pulse after the H_2_bdc pulse in nanocomposite depositions is in between these two cases, indicating that H_2_bdc pulses adds more reactive sites. The H_2_bdc molecule could also react with surface |-Tb-F_*x*_ species from the prior TbF_3_ cycle. This would produce one HF molecule and a significant mass gain due to the much heavier Hbdc^−^ molecule replacing F^−^. As the reaction between H_2_bdc and |-Tb-F_*x*_ is likely slower than with |-Tb-thd_*x*_, this can explain the initial mass decrease followed by a delayed mass increase, which increases above the initial level.

The elemental maps of carbon, terbium and fluorine show the resulting multilayer structure of 200Sm. Probing such small structures is not trivial, in particular with respect to detecting a small amount of Sm^3+^ ions in the TbF_3_ layers. It was attempted to detect the single SmF_3_ layer by EDS or core-loss spectroscopy, but it was not possible to obtain a signal from Sm.

### Luminescence properties

The most striking feature of these nanocomposite films is the realization of aromatic sensitization of strong Sm^3+^ luminescence, whereas direct sensitization by an organic molecule in Sm_2_bdc_3_ shows no luminescence. In fact, the Sm^3+^ luminescence show the same emission spectrum as expected from a YF_3_-type matrix^39^. The enabling component is the bridging ions in the fluoride layer that physically separates the organic sensitizer and the phonon-sensitive Sm^3+^ while still providing energy transfer. This ion must encompass four properties: (1) it must effectively accept the excited state energy from the sensitizer(s), (2) it must not be quenched to a large degree by this sensitizer through its high-energy phonons, red-ox activity or other quenching mechanism, (3) it must not suffer from concentration quenching thus allowing a high enough concentration for long-range energy migration to occur, and (4) the migrating excited state on this ion should be as close as possible in energy to the sensitizer excited state to minimize the energy loss. For bdc^2−^, both Tb^3+^ and Eu^3+^ show strong luminescence^40^, meaning that these two lanthanides are not strongly quenched by the high-energy vibrations on this organic molecule. The higher energy gap in Tb^3+^ makes emission from this ion less quenched by these vibrations than for Eu^3+^ and the quantum yield of Tb^3+^ emissions are higher, making it a better bridge. The term energy gap for lanthanides in this work refers to the gap between the emitting level and the next lower level. If this energy gap is smaller than roughly 5–6 times the maximum phonon energies within interaction distance of the lanthanide, the emitting level will depopulate by creating multiple phonons instead of photons. The excited state of Tb^3+^ is also at higher energies than Eu^3+^, allowing population of higher energy states on the next acceptor ion type. In fact, the emitting ^4^G_5/2_ level of Sm^3+^ lies slightly above the ^5^D_0_ level of Eu^3+^, meaning that Eu^3+^ is a poor bridging ion to Sm^3+^ but can work similarly to Tb^3+^ for other acceptor ions. Thus Tb^3+^ ions is a suited bridge between bdc^2−^ and Eu^3+^.

The next striking feature is the difference in Eu^3+^ emission between 200Eu and Eu_2_bdc_3_, Fig. 3a. Both show similar emission intensity since neither suffer from strong quenching effects in neither the hybrid or fluoride matrices. Eu^3+^ is not strongly quenched by the high-energy organic (C-H) vibrations, thus the main difference between these two materials is the symmetry around Eu^3+^. Directly linking bdc^2−^ and Eu^3+^ produces an amorphous-like emission spectrum while separating them and using TbF_3_ as an energy migration layer produces a YF_3_-like Eu^3+^ emission spectrum^41^. Although the emission spectrum of all lanthanides is affected by symmetry, this effect is particularly important for Eu^3+^ as emission from the non-degenerate ^5^D_0_ level can be used to determine the local site symmetry around the Eu^3+^ ion. Readers interested in the symmetry dependence of Eu^3+^ emission and in particular how this ion is used a probe for local site symmetry are referred to the review article from Binnemans on this topic^42^. Since the energy level positions of Ln^3+^ ions are nearly insensitive to the surrounding matrix, Tb^3+^ can act as a bridge in most types of matrices. Thus, in this example with Eu^3+^, the Eu^3+^ emission spectrum of any matrix can be chosen more or less completely independently of the sensitizer by simply choosing a different Tb^3+^ containing matrix with a different site symmetry than TbF_3_. The Eu^3+^ ion will then have the emission properties of the Tb matrix, independent of the sensitizer species which in turn also can be something else than an organic molecule as long as it can donate energy to Tb^3+^. The only requirement is that the ALD chemistry of this Tb matrix is compatible with the ALD chemistry of the aromatic molecules (or other sensitizer). Interestingly, the 200Eu sample is fundamentally quite similar to the core–shell TbF_3_:Ce^3+^, Eu^3+^ nanoparticle structure by Fischer and Jüstel^15^ where TbF_3_ was used as an energy bridge between Ce^3+^ and Eu^3+^. The main difference being that, instead of Ce^3+^, we have achieved similar sensitization from a completely different material class while maintaining the solid, inorganic multilayered TbF_3_:Ln^3+^ structure.

The situation for Sm^3+^ ions is in some aspects similar to Eu^3+^, but the main difference is that the Sm^3+^ emission is effectively quenched by high-energy phonons due to its lower energy gap. Generally, it is sufficient to consider the maximum phonon energies in the crystalline or amorphous glass matrix, but in this nanocomposite case we have to take the high-energy vibrations of the organic molecule into account and the distance, *R*, between the luminescent lanthanide and these vibrations. The multi-phonon quenching rate decreases with the 1/*R*^6 ^^43^. For Sm^3+^, where the energy gap is small enough to be strongly quenched by these molecules if present in the immediate coordination, the critical factor is whether or not there are any organic molecules within interaction distance of the Sm^3+^ ion. If so, the situation is similar to a lanthanide close to the surface of a nanoparticle being able to interact with the solvent or capping agent. If the molecule is outside interaction distance, Sm^3+^ will behave as if doped into a normal, unsensitized crystalline fluoride matrix. Figure 3c shows the PL intensity of Sm^3+^ and Tb^3+^ emissions from the samples, integrated over the 595–615 and 540–550 nm ranges, respectively, in addition to the lifetime of the 594 nm Sm^3+^ emission. There is a clear correlation between the number of bridging TbF_3_ cycles on one side and the PL intensity and lifetime on the other. Both increase as the distance between the Sm^3+^ ions and high-energy organic vibrations is increased by a thicker TbF_3_ layer. The lifetime is approaching a plateau for 200Sm while the PL intensity reaches a maximum at 100Sm. This difference is likely due to a trade-off between migration through a thicker TbF_3_ layer making it less likely to reach the Sm^3+^ ion and reduced quenching of the Sm^3+^ emission. This is supported by the decay data in Fig. 3d where it is seen that the Sm^3+^ emission of 200Sm has an initial rise during the first 100 µs while 100Sm does not have this rise. The rise indicates that the population of Sm^3+^ ions is slightly delayed. A possible explanation for this is the required energy migration through the TbF_3_ layer. The decay of Eu^3+^ in 200Eu is also shown in Fig. 3d and show the same rise as 200Sm, again indicating a delay caused by the required energy migration. This rise spans about 300 µs, compared to 100 µs for 200Sm. Baur et al. found a similar rise time for Tb_2_Mo_3_O_12_ doped with ≤1% Eu^3+^ when exciting Tb^3+^ ^44^. The rise time decreased with increasing Eu^3+^ concentration and disappeared completely >20%, supporting the explanation that the rise comes from slow migration through the Tb^3+^ network in Tb_2_Mo_3_O_12_.

The difference in rise time between 200Sm and 200Eu can be explained by the differences in energy levels for Sm^3+^ and Eu^3+^ (Fig. 1d). In order to have an energy transfer from Tb^3+^ to Sm^3+^ or Eu^3+^, there must be an overlap between the emission of Tb^3+^ and the absorption of the receiving ion for a Förster resonance energy transfer and matching energy level energies for a Dexter energy transfer. The energy levels of Sm^3+^ are much denser in this range than for Eu^3+^. Thus a possible explanation for the longer rise of 200Eu compared to 200Sm is that the Tb^3+^ → Eu^3+^ energy transfer is slightly slower than the Tb^3+^ → Sm^3+^ energy transfer. There is also the onset of crystallinity for 100Sm compared to 50Sm as seen in Figs. 2c and 3b that causes a change in emission spectra. But from Fig. 3c, there is no particular abrupt change in either the integrated emission intensity or the lifetime between 50Sm and 100Sm, which rather changes gradually with the number of bridging TbF_3_ cycles. Thus the overall emission intensity of Sm^3+^ is dominated by the distance between Sm^3+^ and the organic molecules.

It is difficult to precisely measure the Sm^3+^–molecule distance as we were not able to identify the Sm^3+^ layer in either XRR or STEM due to its characteristics being very similar to Tb^3+^ and the overall doping concentration being very low. However, we can estimate it by assuming that Sm^3+^ is situated in the middle of the TbF_3_ layer, that the organic molecules occupy about 1 nm and using the total film thickness from ellipsometry. These estimates are given above the data points in Fig. 3c. Based on this estimate, the fact that the lifetime is still increasing for 200Sm and that the energy migration efficiency through TbF_3_ seemingly starting to decline at 100Sm, we can conclude that there is a fine thickness range for the bridging layer where it is wide enough to prevent quenching while also being thin enough to enable efficient migration. With ALD, this fine thickness control and optimization of luminescence efficiency are possible.

The third remarkable feature of these films is the realization of emission spectra identical to those of bulk crystalline fluorides while also having very strong optical absorption. The nanocomposite films have only 10 Ln_2_bdc_3_ ALD cycles, resulting in >15% UV absorbance. The absorbance is similar for all *x*Sm samples as they all contain the same amount of terephthalic acid molecules. As the terephthalic acid molecules seemingly does not form a complete and dense layer as the fluoride crystallites are able to growth through these, there is room to further enhance the absorption by a denser packing of these molecules. This can for example be achieved by having more than one Ln_2_bdc_3_ cycle in the organic sensitizer layer. The absorption can also be enhanced by replacing terephthalic acid with a molecule with stronger absorption. Terephthalic acid have a quite strong peak absorption (*ε* = 17.000 M^−1^ cm^−1^), while for example 9,10-anthraquinone (*ε* = 56.800 M^−1^ cm^−1^) and tetraphenylporphyrin (*ε* = 443.000 M^−1^ cm^−1^) absorb 3 and 26 times stronger than terephthalic acid ^35,36^. This means that, through our nanocomposite design, it is possible to design novel luminescent materials that have the emission and conversion properties of a crystalline inorganic material while also having such a strong absorption than only a few tenth or hundreds of nanometre film thickness is necessary for full optical absorption. By comparison, even though the 100Sm and 200Sm samples consists mostly of TbF_3_, the absorption from Tb^3+^ is still below the detection limit of our photospectrometer. A sensitized film can be several orders of magnitude thinner than an unsensitized variant while also having the benefit of a broader and tunable absorption.

## Conclusion

In this work, we have developed a multilayered nanocomposite design for organically sensitized inorganic luminescent materials. This approach has been successful in producing aromatically sensitized Sm^3+^ emission where this emission is normally fully quenched by high-energy organic vibrations. By precisely controlling the separation between Sm^3+^ and the aromatic sensitizer with a TbF_3_ energy migration layer, we were able to both prevent direct Sm^3+^–molecule interactions while maintaining efficient energy migration from sensitizer to Sm^3+^. The films shows the emission and conversion properties of the inorganic component while also having the absorption strength of the organic component, resulting in >15% UV absorption in a 55.9-nm film. This design can be generalized to other luminescent species as well. We obtain orange ^5^D_0_–^7^F_1_ YF_3_-type Eu^3+^ emission from our nanocomposite while direct sensitization gives red ^5^D_0_–^7^F_2_ emission where both materials show the same excitation profile originating from the aromatic sensitizer. This shows that, with our nanocomposite design, we can control the conversion and emission properties of the inorganic layer independently of the sensitizer. The only requirements for this design to be efficient is to have a migration ion like Tb^3+^ or Eu^3+^ that can accept energy from the chosen sensitizer and donate it to any type of luminescent acceptor ion and that the ALD chemistries of the sensitizer and inorganic layer is compatible. The ALD literature show that there is a wide variety of examples combining very different materials type due to ALD’s unique chemical flexibility coming from the low temperature and layer-by-layer deposition. With the chemical flexibility of ALD, this multilayer nanocomposite design can bridge the gap to strongly sensitized multi-component luminescent systems like upconversion and downconversion.

## Methods

### Sample structure and design

The general sample structure is illustrated in Fig. 1a. It consists of repeating supercycles, where each supercycle consist of a single aromatic acid pulse, followed by a varying number of TbF_3_ cycles where the cycle in the centre is replaced by an SmF_3_ or EuF_3_ cycle. This produces a TbF_3_ layer with a single, atomically thin dopant layer of Sm^3+^ or Eu^3+^ in the middle. Figure 1b illustrates the energy levels through the multilayered structure, showing broadband UV absorption in the aromatic terephthalic acid molecules (Ar), transfer to Tb^3+^ and migration through the TbF_3_ layer before final transfer onto Sm^3+^ and subsequent emission. The major variable in this design is the number of TbF_3_ cycles that separates the aromatic acid and the second lanthanide (Sm or Eu). Sm^3+^ is strongly quenched by high-energy vibrations on organic species, so the emission intensity of Sm^3+^ also works as an efficient probe for the Sm^3+^–acid distance to detect if the distance is sufficient to prevent direct Sm^3+^–acid interactions. We have chosen to use the less explored NH_4_F fluorine source rather than the already established Ln(thd)_3_+TiF_4_ route because TiF_4_ is known to leave small amounts of Ti^4+^ impurities^22,45^. Due to the energy migration in the TbF_3_ layer, even small amounts of Ti^4+^ can completely quench the excited state due to the low-energy Tb^4+^–Ti^3+^ charge transfer state ^46^.

The films were deposited in an F-120 research-type ALD reactor (ASM Microchemistry Ltd) at 250 °C. Ln-β-diketonate Ln(thd)_3_ (Strem Chemicals, >99.9%, Ln = Sm, Eu, Tb) were used as lanthanide precursors, while terephthalic acid (H_2_bdc) and NH_4_F were used as anion precursors for metal organic hybrid cycles and LnF_3_ cycles. Nitrogen was used as carrier and purge gas, supplied from gas bottles (AGA, 99.999%). p-type Si(100) substrates were used for all depositions. In addition, 0.5 × 4 cm^2^ Si(100) strips were placed some 8 cm apart in the gas inlet and exhaust sides of the deposition chamber to monitor thickness gradients. The native oxide on the silicon substrates was not removed. All substrates were dry wiped and dust was removed using pressurized air prior to the ozone treatment. The sublimation temperature used for Ln(thd)_3_ was 150, 145 and 140 °C for Sm, Eu and Tb, respectively, 210 °C for H_2_bdc and 85 °C for NH_4_F. Pulse parameters for LnF_3_ and Ln_2_bdc_3_ cycles were 1.5/1/5/1 s for Ln(thd)_3_/purge/anion/purge, where anion is either NH_4_F or H_2_bdc.

Two sets of samples were deposited. Those named Ln-bdc are 100–500 cycles of Ln_2_bdc_3_. These were deposited to investigate the bdc → Ln^3+^ energy transfer, Ln^3+^ luminescence and quenching of this emission. The second set of samples all consist of 10 supercycles of 1 Tb_2_bdc_3_ cycle, *x* TbF_3_ cycles, 1 LnF_3_ cycle (Ln = Sm or Eu) and *x* TbF_3_ cycles. These samples will have a similar total amount of organic molecules and emitting Eu^3+^/Sm^3+^ ions, and thus similar absorption and number of emissive sites, simplifying the comparison of their luminescence. The major difference between these samples is the *x* number of cycles separating the bdc molecule and the non-Tb Ln^3+^ ion. The samples are named *x*Ln, i.e. 50Sm means a sample of 10 supercycles of (1 Tb_2_bdc_3_, 50 TbF_3_, 1 SmF_3_, 50 TbF_3_).

In situ QCM analyses were conducted using a 6-MHz AT cut quartz crystal. The crystal was mounted in a home-made holder and was used to monitor the mass increase during the deposition in order to determine saturation conditions with respect to pulse and purge parameters. The standard parameters for both TbF_3_ and Tb_2_bdc_3_ cycles were 1.5/1/3/1 s for all QCM experiments. Only one parameter was changed at a time. The signal was recorded using a Colnatec Eon-LT and processed by averaging over 16 consecutive ALD cycles. The temperature was stabilized for 90 min before any experiments were conducted to ensure a reliable response from the QCM crystals.

### Structural and optical characterization

The crystallinity of the samples was determined with a Bruker D8 Discovery X-ray diffractometer (XRD), using CuKα1 radiation and a Ge(111) monochromator. XRR was measured on a PanAlytical Empyrian instrument using CuKα1 radiation without monochromator, while data were analysed using the PanAlyticals X’Pert Reflectivity software. Film thickness and refractive index were determined with a J. A. Woollam alpha-SE spectroscopic ellipsometer in the 380–900 nm range. The ellipsometry data were modelled using a Cauchy model.

PL and PLE measurements were done using two different set-ups. A 280 nm diode and an USB2000+ spectrometer from OceanOptics were used for recording the PL spectra in Fig. 3a, while an Edinburgh Instruments FLS920 fluorescence spectrometer with a 450 W Xe lamp as excitation source and a Hamamatsu R928 PMT for detection was used for the PLE spectra in Fig. 3a and the PL spectra in Fig. 3b, c. PL decay measurements were performed with the third harmonic of a Nd:YAG laser as excitation source of the OpotekHE 355 II with an ~10 ns pulse width and a repetition rate of 20 Hz. The decay was recorded with MultiChannelScaling function incorporated in the Edinburgh Instruments Spectrofluometer. Absorbance data were obtained by combining transmittance and reflectance measurements in a Shimadzu UV3600 photospectrometer using an integrating sphere.

STEM investigations of the samples were conducted after standard sample preparation techniques, by cutting, manual grinding and polishing. Final thinning was done with a Gatan PIPS II ion mill using argon ions, with gradually decreasing voltages and angles and increasing polishing time. Plasma cleaning with a Fishione Model 1020 for ca. 5 min was applied prior to the TEM experiments. STEM was performed with a monochromated FEI Titan G2 60-300 kV TEM, equipped with a CEOS probe-corrector. The microscope was operated at 300 kV with a convergence angle of 20 mrad, where HAADF imaging was done with collection angles 40–200 mrad.

## Data Availability

The data presented in this study are available from the corresponding author upon reasonable request.
